# PPARγ Agonists: Emergent Therapy in Endometriosis

**DOI:** 10.3390/ph14060543

**Published:** 2021-06-06

**Authors:** Alexandre Vallée, Jean-Noël Vallée, Alain Le Blanche, Yves Lecarpentier

**Affiliations:** 1 Department of Clinical Research and Innovation (DRCI), Foch Hospital, 92150 Suresnes, France; 2 Centre Hospitalier Universitaire (CHU) Amiens Picardie, Université Picardie Jules Verne (UPJV), 80000 Amiens, France; valleejn@gmail.com; 3 DACTIM-Mis, Laboratoire de Mathématiques et Applications (LMA), UMR CNRS 7348, Université de Poitiers, 86000 Poitiers, France; 4 Laboratoire CeRSM (EA-2931), UPL, Université Paris Nanterre, F92000 Nanterre, France; alain.le.blanche@gmail.com; 5 Hôpital René-Dubos de Pontoise and Université de Versailles-Saint-Quentin, Simone Veil UFR des Sciences de la Santé, 78180 Montigny-le-Bretonneux, France; 6 Centre de Recherche Clinique, Grand Hôpital de l’Est Francilien (GHEF), 77100 Meaux, France; yves.c.lecarpentier@gmail.com

**Keywords:** endometriosis, PPARγ agonists, inflammation, angiogenesis, apoptosis, invasion, adhesion

## Abstract

Endometriosis is one of the major gynecological diseases of reproductive-age women. This disease is characterized by the presence of glands and stroma outside the uterine cavity. Several studies have shown the major role of inflammation, angiogenesis, adhesion and invasion, and apoptosis in endometriotic lesions. Nevertheless, the mechanisms underlying endometriotic mechanisms still remain unclear and therapies are not currently efficient. The introduction of new agents can be effective by improving the condition of patients. PPARγ ligands can directly modulate these pathways in endometriosis. However, data in humans remain low. Thus, the purpose of this review is to summarize the potential actions of PPARγ agonists in endometriosis by acting on inflammation, angiogenesis, invasion, adhesion, and apoptosis.

## 1. Introduction

Endometriosis is a gynecological disorder characterized by the presence of glands and stroma outside the uterine cavity [1]. Six to ten percent of reproductive-age women are affected by this disease. The main symptoms of this disease are infertility and pelvic pain, with over 10% of women affected [2]. Other symptoms are listed, such as dysuria, dyspareunia, dysmenorrhea, and irregular uterine bleeding [3,4]. Nevertheless, the diagnosis of this disease remains uncommon [5]. Lesions observed in endometriosis are for the main part observed in the ovaries, fallopian tubes, the ligaments of the uterus, the cervical-vaginal area, the abdominal wall and umbilicus, the urinary tract, and the rectum [6,7]. Several factors have been observed as initiators of endometriosis, including immune, genetic, endocrine, and environmental markers [8,9]. However, the mechanisms of endometriosis initiation and development remain unclear.

Recent studies have shown that inflammation processes are an important factor in endometriosis through their action on angiogenesis, apoptosis, and cell proliferation [1]. The pro-inflammatory hypothesis was reported in several studies showing that peritoneal fluid of endometriotic patients presents high levels of activated macrophages, angiogenic markers, cytokines, and growth factors [10,11]. Moreover, inflammatory cells may be the initiators of endometriotic processes [12].

Hormone therapy, medication, and surgery are used to eradicate the symptoms in endometriotic patients. Pain-relieving, non-steroidal anti-inflammatory drugs, aromatase inhibitors, progestins, combined estrogen–progestin therapy, and selective progesterone receptor modulators are the main common recommended therapies [7,13]. Nevertheless, the mechanisms underlying this disease are still unclear, and therapies are not currently efficient.

The introduction of new agents can be effective by improving the condition of patients [14]. PPARγ ligands can directly modulate inflammation, metabolic processes, fibrosis, angiogenesis, and several other pathways [15,16,17]. In endometrial stromal cells, both rosiglitazone and pioglitazone reduce inflammation process by acting on IL-6 secretion [18]. Moreover, other PPARγ agonists can act by reducing angiogenesis [19], invasion and adhesion [20], and apoptosis [21].

Thus, the purpose of this review is to summarize the potential actions of PPARγ agonists in endometriosis by acting on inflammation, angiogenesis, invasion and adhesion, and apoptosis.

## 2. PPARγ

PPARs (peroxisome proliferator-activated receptors) belong to the superfamily of nuclear hormone receptors and call for their activation, which is controlled by the peroxisome proliferators. There are three subtypes of PPARs (PPARα, PPARβ, and PPARγ). These subtypes are expressed differently in tissues. PPARγ is mainly expressed in adipose tissue [22]. PPARs consist of four domains (A/B, C, D, and E/F). The A/B region, localized at the N end of the receptor protein, is the active functional region, differs among the PPARs subtypes, and is independent of ligands. Region C is the DNA-binding domain containing two zinc finger structures. Region D is the hinge domain. Region E/F, localized at the end of C, is the ligand-binding domain and contains a ligand-dependent transcriptional activation functional region [23]. The PPARγ gene can be transcribed into different PPARγ mRNAs and translated into two isoforms (PPAR γ1 and PPAR γ2) [24]. PPARs are ligand-activated transcription factors that bind PPRE (PPAR-response elements). In the nucleus, PPARs form a heterodimer with the retinoid X receptor (RXR) [25]. They are composed of a ligand-binding domain that interacts with a DNA-binding domain to modulate it [26]. PPARs are involved in numerous pathophysiological processes, such as cell differentiation, protein metabolism, lipid metabolism, carcinogenesis [27,28], adipocyte differentiation, insulin sensitivity, and inflammation [29,30]. Among the many biological responses involved, PPARγ has a corresponding function by controlling the expression of several pathways, such as JAK-STAT, NF-kB, nuclear factor of activated T cell, AP-1, PI3K, leptin, and adiponectin [31].

PPARγ ligands can be synthetic or natural. PPARγ ligands have hypoglycemic and hypocholesterolemic roles, such as glitazones, which have been used in the treatment of type 2 diabetes [32]. PPARγ ligands, such as thiazolidinediones (TZDs), can also decrease inflammatory activity [32]. Natural ligands include prostaglandins and unsaturated fatty acids [33]. PPARγ ligands can be synthetic or natural. Natural ligands include prostaglandins and unsaturated fatty acids. Moreover, PPARγ ligands, such as thiazolidinediones, can directly decrease inflammatory activity [17], the fibrosis process [34], and lung inflammation [35]. PPARγ agonists have an antiangiogenic role observed in several organs [36,37]. Indeed, PPARγ activation leads to the inhibition of the expression of vascular endothelial growth factor (VEGF), a proangiogenic factor [38]. The expression of PPARγ is observed in the choriocapillaris, choroidal endothelial cells, retinal endothelial cells, and retinal pigmented epithelium [39]. Several animal models have shown the interest of PPARγ agonists in endometriosis [40]. Ciglitazone and rosiglitazone can both prevent and treat endometriosis in a rodent model of the disease [41,42,43,44]. A randomized study also confirmed the effectiveness of rosiglitazone in baboons [45]. Nevertheless, data in humans remain low [21].

## 3. PPARγ Agonists Actions on the Pathophysiology of Endometriosis

### 3.1. Inflammation

Inflammation presents a major role in the process of endometriosis [46]. The cascade of the different inflammatory factors stimulates several markers, including metalloproteinases, prostaglandins, cytokines, and chemokines [6]. These markers are stimulated in the peritoneal serum of endometriosis patients and in the endometrium [47,48,49]. However, normal cells of the endometrium are not affected by this mechanism [50]. Interlieukin-10 (IL-10, IL-6, IL-8, COX2 (cyclooxygenase-2)), VEGF, and tumor necrosis factor α (TNF-α) are upregulated in the peritoneal fluid of endometriosis [49,51]. The stroma of the endometrium are correlated with the process of adhesion of extracellular matrix proteins when IL-8 and matrix metalloproteinase (MMP) have been upregulated [52,53,54,55]. Furthermore, endometriosis is associated with the activation of the NF-κB pathway leading to enhance cell growth, proliferation, and apoptosis during the process of endometriosis [56].

The cyclooxygenase-2 (COX-2)/prostaglandin E2 (PGE2) pathway is involved during endometriosis. Pelvic inflammation is closely related to the activation of peritoneal immune cells, such as macrophages [57,58,59]. Women with endometriosis show an increased number of macrophages with dysfunctional phenotype. Peritoneal macrophages have reduced phagocytic capacity due to lower levels and activity of MMP9, which is required for extracellular matrix degradation and is regulated by PGE2 [60]. Elevation of COX-2 expression has a main role in the initiation and development of endometriosis [61,62]. The overexpression of COX-2 contributes to the increase of PGE2 in endometriosis [63] and participates in the control of ectopic implantation and endometrium growth, angiogenesis, and immunosuppression [64]. Thus, PGE2 appears to be one of the main controllers of the immune response in endometriosis [65]. The endometrial glandular epithelium is the localization of COX-2 production. This production varies according to the menstrual cycle. Expression of COX-2 is low in the early proliferative stage and gradually increases thereafter to reach a maximum at the secretory stage [66]. The expression of COX-2 is higher in women affected by endometriosis in the endometrial stroma and glandular epithelium than in non-affected women [67,68,69,70]. Nevertheless, the COX-2 levels vary throughout the menstrual cycle regardless of whether its expression is affected [67]. During the proliferative and secretory phase, in the eutopic endometrium and in ovarian endometriotic tissue, COX-2 expression is higher in endometriosis women compared with healthy women [71]. NF-κB pathway can directly activate the COX-2/PGE2 to increase the production of estrogen in the endometrium [72].

In endometrial stromal cells, both rosiglitazone and pioglitazone can reduce the inflammation process by acting on IL-6 secretion [18] (Table 1). Glitazone can target Il-6 to decrease the activity of the STAT3 pathway [73,74]. Moreover, TZDs can diminish the expression of numerous inflammatory factors, and TZDs could be used as treatment for pain management in endometriosis [44,75,76].

### 3.2. Angiogenesis

Angiogenesis is marked by the formation of new capillaries by proliferative and migrative phenomenon of preexisting differentiated endothelial cells. The angiogenesis process operates in both embryonic initiation and postnatal life [86,87]. Angiogenesis is characterized by the dysfunction of the vessel basement membrane and the surrounding extracellular matrix (ECM) [88]. The MMP enzyme family degrade components of ECM by collagenases, gelatinases, stromelysins, and membrane-associated MMPs. Gelatinase-A (MMP-2) and gelatinase (MMP-9) are present in blood vessels. MMP-2 and MMP-9 have synergistic effects on basement membrane degradation [89]. The angiogenesis process is observed in endometriosis with several angiogenic factors involved, such as IL-8, VEGFA/C, and angiogenin in both clinic specimens and animal models [90,91,92].

Several signaling pathways are implicated in angiogenesis initiation [93]. The dysregulation of growth factors has a main function in angiogenesis [94]. VEGF can be activated by physiological stimulators, such as inflammation and hypoxia processes [95,96]. Hypoxia-inducible factor 1 α (HIF-1α)/VEGF signaling leads to the activation of different processes, such as proliferation and migration of endothelial cells [97]. Several stages can define angiogenesis: blood vessel breakdown, basement membrane degradation, a surrounding extracellular matrix (ECM), endothelial cells migration, and new blood vessel formation [98]. From existing vessels, novel blood vessels are generated through the dissolution of aspects of native vessels. Angiopoietin-1 and 2 (ANG-1 and ANG-2) are main endothelial growth factors that operate by the tyrosine kinase with immunoglobulin and EGF homology domains (TIE-2) receptor tyrosine kinase (RTK) expressed in endothelial cells. Under physiological conditions, ANG-1 binds to TIE-2, leading to the association between pericytes and endothelial cells to stabilize the vasculature [99,100]. ANG-1 acts as an activator ligand for TIE-2, whereas ANG-2 inhibits the phosphorylation of TIE-2, even in the presence of ANG-1 [101,102]. TIE-2 is a major marker of the physiological vascular initiation and of mature vasculature homeostasis [103]. Destabilization of the blood vessels’ structure is induced by ANG-2, which acts as an antagonist of TIE-2 phosphorylation [102,104]. Thus, VEGF expression leads to the proliferation and migration of endothelial cells in the presence of ANG-2 to participate in the stimulation of new blood vessel growth [105]. AT1R stimulation involves the increase of VEGF expression to activate angiogenesis [106]. Moreover, AT1R activates inflammation by increasing the expression of leukocytic and endothelial adhesion molecules [107].

In vitro studies have shown interest in using anti-VEGF factors to decrease the growth of endometriosis without impact on ovarian function [108]. The stimulation of VEGF leads to the activation of the phosphatidylinositol 3-kinase-protein kinase B (PI3K/Akt) pathway [109]. This stimulated signaling can initiate the angiogenesis process and can participate in the decrease of apoptosis [110]. Through the stimulation of both hypoxia inducible factor 1 alpha (HIF-1α) and cyclin D1, the PI3K/Akt pathway involves angiogenesis without hypoxia [110,111]. The decrease in NME1 gene expression in the endometrium stimulates both the PI3K/Akt pathway and the expression of VEGF and IL-8 to induce production of new vascular cells in ectopic endometrial lesions [112]. In endometriosis, the stimulation of the PI3K/Akt pathway is associated with NOS expression and oxidative stress [113]. By maintaining the fibrotic environment of endometriosis, the PI3K/Akt pathway stimulates the ERK pathway [114]. Furthermore, in endometriosis, the PI3K/Akt pathway stimulates the NF-κB pathway, a major stimulator of VEGF, leading to cell proliferation and angiogenesis [115].

Several animal studies have shown that the use of PPARγ agonists lead to the prevention of the development of endometriotic-like lesions [21,41,42,43,45,77] (Table 1). TZDs downregulate the proliferation of endothelial cells and lead to the reduction of vasculature lesions by inhibiting VEGF expression [44,76,116,117]. The stimulation of PPARγ expression is associated with the reduction of AT1R proteins in endometriotic lesions leading to a decrease in the density of CD31-positive micro-vessels and to the reduction of immune cells [78].

Telmisartan, a PPARγ agonist possessing anti-atherogenic properties, can block AT1R in endometrial stromal cells [19]. Thus, the AT1R antagonism of telmisartan can downregulate the choroidal inflammation and neovascularization [118] and can prevent hepatocarcinogenesis through the suppression of hepatic blood vessel formation [119]. The anti-angiogenic action of telmisartan is modulated by the stimulation of PPARγ [78,120]. Furthermore, rosiglitazone can diminish endothelial cell proliferation and migration and can decrease the expression of VEGF in human umbilical vein endothelial cells [79]. Pioglitazone and rosiglitazone can decrease the activation of basic fibroblast growth factor (bFGF)- and VEGF-induced blood vessel formation in the chorioallantois membrane model [80].

### 3.3. Adhesion and Invasion

Molecules adhesion increases the attachment of endometrial-like tissues to ectopic sites [121]. Several findings have shown that ectopic endometriotic cells possess the ability to invade their surrounding environment and that they have the potential to metastasize in lymph nodes and in the abdominal cavity [122].

An increase in the production of MMP, including MMP-1,2,3,9,11, ICAM-1, integrins, and cadherins, has been observed in the peritoneum of endometriotic women. These molecules have a major function in tissue attachment, in the invasion of ectopic lesions, and in implant progression and angiogenesis [123,124]. Thus, MMPs are involved in several reproductive mechanisms, such as ovulation, menstruation, and embryo implantation [125,126]. The expression of endometrial MMPs is low in the proliferative stage, declines in the early secretory stage, but increases in the late secretory stage. One of the main inhibitors of MMPs is progesterone. Progesterone controls many different hormones, growth factors, and cytokines. MMPs can modulate the expression of progesterone by controlling the plasminogen activator (PAI) pathway [127,128,129]. Retinoic acid and transforming growth factor-β (TGF-β) both increase the expression of tissue inhibitors of metalloproteinases (TIMPs), allowing MMP activity to mediate the maintenance and survival of lesions [130].

Moreover, PGE2 acts through G protein-coupled receptors and modulates many cellular pathways. In endometriosis, these G protein-coupled receptors are defined by three subtypes of the PGE receptor (EP2, EP3, and EP4) [131]. Previous findings have shown that EP receptors intracellularly transactivate the MAPK, Wnt, and PI3K/Akt pathways, leading to the modulation of cell apoptosis, proliferation, invasion, migration, angiogenesis, pain, and immunomodulation [132,133]. The administration of COX-2 inhibitors is associated with the decrease in survival, migration, and invasion of endometriotic cells, resulting in a decrease of PGE2 production [134,135]. The inhibition of COX-2 in endometriotic cells is controlled by MMP-2 and MMP-9 [136]. Nevertheless, the underlying molecular processes of COX-2 inhibitors remain unclear [64].

PPARγ activation is associated with a reduction of invasion by acting on LP9 cells and through the co-adhesion molecule (CAM) on peritoneal mesothelial cells [81] (Table 1). Previous findings have explored the potential effects of PPARγ agonists on the inhibition of CAM expression in inflammatory processes. Pioglitazone can influence monocyte–endothelial cell binding by inhibiting vascular cell adhesion molecule-1 (VCAM-1) expression on activated endothelial cells [82]. PPARγ agonists can suppress the vascular adhesion molecule expression in endometriosis [20]. PPARγ agonists can decrease the expression of ICAM-1 in lung epithelial cells, leading to the b2-integrin-mediated adhesion of monocytic effector cells to monolayers of these respiratory syncytial virus-infected human lung cells [83]. Moreover, rosiglitazone can reduce proliferation, apoptosis, and invasion in in vitro endometrial tissues by acting on the promoters of the MAT2A gene [84].

### 3.4. Apoptosis

The maintenance of tissue homeostasis is modulated by cell death. An imbalance is observed between cell apoptosis and proliferation to maintain this homeostasis against cell disorders. Many findings have shown that apoptosis is upregulated during the menstrual cycle to retain cell homeostasis and to remove aged cells from the functional layer of the endometrium [137,138]. In endometriosis, the diminution in cell death may be the cause of the development of this disease [139,140]. The proportion of cell apoptosis is diminished in endometrial cells [141]. Furthermore, in endometriosis, the stimulation of the NF-κB pathway is correlated with both proliferation and apoptosis [142,143].

The PI3K/Akt pathway is stimulated in endometriotic cells [144] and increases the apoptosis process [145]. A vicious circle is observed between NF-κB and PI3K/Akt pathways to increase the activation of cell apoptosis [146]. The NF-κB pathway decreases the antiapoptotic function of the PI3K/Akt pathway [147]. Xlinked inhibitor of apoptosis protein (XIAP) acts as caspase-3 and caspase-9 inhibitors and modulates the BAX/cytochrome c pathway to involve apoptosis by diminishing the expression of caspase-9 [148]. The PI3K/Akt pathway leads to the activation of XIAP and B cell lymphoma extra-large (BclxL) expressions. Furthermore, Bcl-xL is stimulated by the PI3K/Akt pathway [149,150]. In ectopic endometriotic tissues, Bcl-2 exerts many modulatory functions in both cellular apoptosis and proliferation [151]. Bcl2 can lead to antiapoptotic features [152]. In physiological conditions, the stimulation of extracellular signal-regulated kinases 1 and 2 (ERK1/2) increases the proliferation of cells and then promotes angiogenesis [153,154]. ERK1/2 activation decreases Bcl2 expression, decreasing mitochondrial-dependent cell death [155]. The PI3K/Akt and MAPK pathways both stimulate the process of anti-apoptosis in endometriosis [144,156]. An opposing interplay is observed between the ERK and PI3K/Akt pathways [157,158]. A decrease in the PI3K/Akt pathway is associated with the stimulation of the ERK pathway [159]. The reciprocity is verifiable [160]. Many findings have shown an interest in co-targeting these two signaling pathways in endometriotic cells [161].

Inhibition of EP2 and EP4, two receptors of PGE2, can facilitate the interactions between antiapoptotic proteins, such as Bcl-2 and Bcl-XX, and proapoptotic proteins, such as Bax and Bad, to increase cytochrome c release and to then activate the caspase-3 pathway [162]. Moreover, the inhibition of both COX-2 and PGE2 is associated with decrease in proliferation in endometriotic epithelial and stromal cells [135]. Thus, the administration of COX-2 inhibitors to the ectopic and eutopic endometrium can participate in decreasing proliferation, growth, and then to increasing apoptosis [163].

PPARγ activation is associated with apoptosis and cell cycle regulation in human endometriotic epithelial and stromal cells [21]. Rosiglitazone can decrease endometriotic implant growth, cell proliferation, and vascularization, and it stimulates the process of apoptosis in the mouse model of endometriosis [44] (Table 1). Rosiglitazone, ciglitazone, or pioglitazone can decrease the growth of implants in a mouse model of endometriosis [41,42,43] and can diminish postsurgical adhesions with endometriotic lesions in a chimeric mouse model [85]. The role of estrogen in the growth of endometriosis is well known. P450 aromatase can catalyze the last stages of the biosynthesis of estrogen from androgens in two ways: (a) from androstenedione into estrone and (b) from testosterone into estradiol [164,165,166,167]. The P450 aromatase protein is highly expressed in endometriosis [165]. In endometriotic stromal cells, PGE2 stimulates the expression P450 aromatase by EP2 [166,167]. TZDs, PPARγ agonists, can diminish the expression of P450 aromatase in human breast tissue and breast cancer [168,169] as well as in ovarian granulosa cells [170]. The function for PGE2 is well-known in the initiation of endometriosis [162,163,171]. Recent findings have shown that PGE2, via EP2 and EP4, can stimulate the cAMP/PKA/CREB pathway, enhancing P450 aromatase transcription and increasing the activity of aromatase [168]. Stimulation of PPARγ by pioglitazone can downregulate P450 aromatase expression through the induction of BRCA1 and through the inhibition of the PGE2/cAMP/KA pathway in breast cancer [168].

## 4. Conclusions

PPARγ agonists can decrease inflammation, angiogenesis, invasion and adhesion, and can induce apoptosis in endometrial lesions. Nevertheless, these actions are for the main part observed in animals and remain hypothetical in women. However, the use of natural PPARγ ligands could be interesting in dietary prevention and disease management for women. Nevertheless, the limited number of studies focusing on the different interactions of PPARγ agonists in endometriosis restricts its clear and immediate use as a therapeutic strategy. Future clinical trials are needed to better investigate and highlight the role of PPARγ agonists in endometriosis.

## Figures and Tables

**Table 1:** Mechanisms by which PPARγ agonists modulate inflammation, angiogenesis, adhesion and invasion, and apoptosis in endometriosis.

Target	PPARγ agonist	Target	Actions	Model	References
Inflammation	Rosiglitazone	-	Decreased symptom severity scale and pain	Women	[75]
	Rosiglitazone	-	Diminution of implant volume, cell proliferation, apoptosis, and inflammation	Female BALB/c mice	[44]
	Rosiglitazone	-	Diminution of inflammation	Endometriotic stromal cells	[76]
Angiogenesis	Ciglitazone	Decrease VEGF	Decreased the size of ectopic uterine tissues and the mean explant wet weight	Rat model of endometriosis	[41]
	Rosiglitazone	Decrease VEGF	Endometriotic lesions were statistically significantly lower in rosiglitazone-treated baboons when compared with the placebo group	Female baboons	[45]
	Pioglitazone	Decrease VEGF	The surface area and volume of endometriotic lesions were significantly lower in pioglitazone-treated baboons than the placebo group	Female baboons	[77]
	Ciglitazone	Decrease PGE2 Decrease P450 aromatase	Inhibition growth cells and cell proliferation	Endometriotic epithelial cells	[21]
	Pioglitazone	reduction of AT1R proteins	Decrease in density of CD31-positive micro-vessels	Murine endometriosis-like lesions	[78]
	Rosiglitazone	Decrease VEGF	Reduction of endothelial cell proliferation and migration	Human umbilical vein endothelial cells	[79]
	Pioglitazone and rosiglitazone	Decrease bFGF and VEGF	Reduction in blood vessel formation	Chorioallantois membrane model	[80]
Adhesion and Invasion	Ciglitazone	Decrease CAM	Reduction of invasion	LP9 cells	[81]
	Pioglitazone	Decrease VCAM-1	Inhibition of vascular cell adhesion	Endothelial cells	[82]
	15d-PGJ2, Ciglitazone, Troglitazone	Decrease ICAM-1	b2-integrin-mediated adhesion	Lung epithelial human cells	[83]
	Rosiglitazone	Modulation promoters of MAT2A gene	Reduction of proliferation, apoptosis, and invasion	In vitro endometrial lesions	[84]
Apoptosis	Rosiglitazone	-	Endometriotic implant growth	Female BALB/c mice	[44]
	Rosiglitazone	-	Endometriotic implant growth	Rat model	[42]
	Rosiglitazone	-	Decrease in height and spherical volumes	Rat model	[43]
	Pioglitazone	-	Diminution of postsurgical adhesions	Chimeric mouse model	[85]

## Data Availability

No new data were created or analyzed in this study. Data sharing is not applicable to this article.
